# Prospects of liquid biopsy in the prognosis and clinical management of gastrointestinal cancers

**DOI:** 10.3389/fmolb.2024.1385238

**Published:** 2024-05-06

**Authors:** Deepankar Mondal, Sapnita Shinde, Vibha Sinha, Vineeta Dixit, Souvik Paul, Rakesh Kumar Gupta, Suresh Thakur, Naveen Kumar Vishvakarma, Dhananjay Shukla

**Affiliations:** ^1^ Department of Biotechnology, Guru Ghasidas Vishwavidyalaya, Bilaspur, Chhattisgarh, India; ^2^ Department of Botany, Sri Sadguru Jagjit Singh Namdhari College, Garhwa, Jharkhand, India; ^3^ Department of Surgical Gastroenterology, All India Institute of Medical Sciences, Raipur, Chhattisgarh, India; ^4^ Department of Pathology and Lab Medicine, All India Institute of Medical Sciences, Raipur, Chhattisgarh, India; ^5^ Trivitron Healthcare Pvt Ltd., Chennai, India

**Keywords:** colorectal cancer, gastrointestinal cancers, liquid biopsy, prognosis, circulating tumor cells, clinical applications

## Abstract

Gastrointestinal (GI) cancers account for one-fourth of the global cancer incidence and are incriminated to cause one-third of cancer-related deaths. GI cancer includes esophageal, gastric, liver, pancreatic, and colorectal cancers, mostly diagnosed at advanced stages due to a lack of accurate markers for early stages. The invasiveness of diagnostic methods like colonoscopy for solid biopsy reduces patient compliance as it cannot be frequently used to screen patients. Therefore, minimally invasive approaches like liquid biopsy may be explored for screening and early identification of gastrointestinal cancers. Liquid biopsy involves the qualitative and quantitative determination of certain cancer-specific biomarkers in body fluids such as blood, serum, saliva, and urine to predict disease progression, therapeutic tolerance, toxicities, and recurrence by evaluating minimal residual disease and its correlation with other clinical features. In this review, we deliberate upon various tumor-specific cellular and molecular entities such as circulating tumor cells (CTCs), tumor-educated platelets (TEPs), circulating tumor DNA (ctDNA), cell-free DNA (cfDNA), exosomes, and exosome-derived biomolecules and cite recent advances pertaining to their use in predicting disease progression, therapy response, or risk of relapse. We also discuss the technical challenges associated with translating liquid biopsy into clinical settings for various clinical applications in gastrointestinal cancers.

## 1 Introduction

Gastrointestinal (GI) cancers, encompassing colorectal, stomach, esophageal, liver, and pancreatic cancers, exhibit varying global incidences and mortality rates (Siegel et al., 2023a). A recent estimate reports that gastrointestinal cancers contribute to one in three cancer incidences and one in three cancer-related deaths globally and are expected to increase in the coming years (Sathishkumar et al., 2022; Kayali et al., 2023). Gastrointestinal tract cancers also result in the impairment of almost all quality of life (QOL) parameters, and therefore, it is essential to enhance cancer care and clinical outcomes for these cancer types (Dalhammar et al., 2022; Siddiqui et al., 2023). Among all GI tract cancers, colorectal cancer (CRC) stands as one of the most prevalent cancers worldwide, with mortality rates gradually decreasing due to improved treatments and early detection (Siegel et al., 2023b). Stomach or gastric cancer (GC), while experiencing a decrease in incidence in many regions, still presents significant mortality rates influenced by the stage of diagnosis and treatment accessibility (Iwu and Iwu-Jaja, 2023). The global incidence of esophageal cancer varies, with mortality rates often high due to late-stage detection (Joseph et al., 2022). Liver cancer (hepatocellular carcinoma, HCC) incidence is linked to factors like viral infections and lifestyle choices, resulting in high mortality rates, often due to late-stage diagnosis (Rattanasupar et al., 2021). Pancreatic cancer, which is challenging to treat and frequently diagnosed at advanced stages, registers lower survival rates among gastrointestinal cancers (Li C. et al., 2022). Efforts toward early detection through screenings, lifestyle modifications, and advancements in treatment remain pivotal in mitigating mortality rates across these cancers (Kayali et al., 2023).

Colorectal cancer is a major global health concern due to its high incidence rates and considerable fatality figures. It is rated as the third most typical cancer among those who are diagnosed in both men and women worldwide. Recent data show that CRC had a global incidence of 1.93 million and caused approximately 0.93 million deaths in 2020, which is expected to increase to 3.2 million new cases in 2040 (Siegel et al., 2023a; Siegel et al., 2023b). In addition to this, lower survival statistics describe the difficult environment that patients with colorectal cancer and clinicians must navigate. Despite improvements in medical treatment, this type of cancer frequently poses a significant barrier to increased life expectancy. Multiple variables, including late-stage detection, aggressive tumor behavior, and a lack of effective treatments for advanced patients, contribute to reduced survival rates (Siegel et al., 2023a; Siddiqui et al., 2023). The American Cancer Society reports that the colorectal cancer 5-year survival rate varies greatly depending on the stage at diagnosis. Patients with localized tumors (stages I–II) have a 5-year survival rate of nearly 90%, while those with stage III have a survival rate of nearly 72% compared to a much lower rate for those with stage IV and distant metastases, which is around 14% (Padilla-Ruiz et al., 2022; Siegel et al., 2023b). This discrepancy underlines the crucial significance of early detection using methods that can overcome difficulties presented by conventional diagnostic approaches and enhance the general prognosis for those affected by these illnesses. In the search for a minimally invasive and extremely sensitive method for detection, liquid biopsy has emerged as a new strategy in the diagnosis of many malignancies.

## 2 Prognostic cues captured through liquid biopsy have useful clinical importance

**FIGURE 1:**
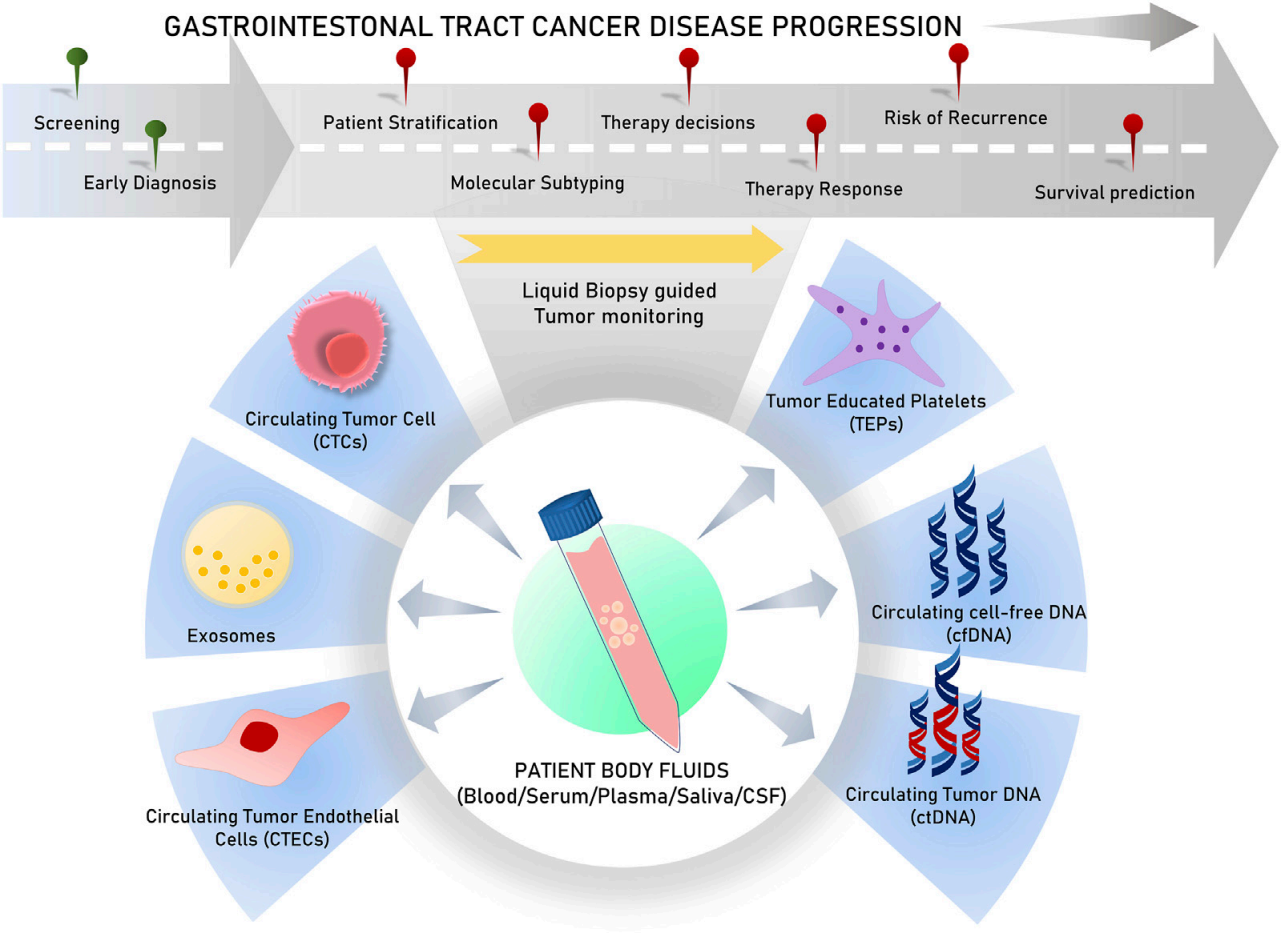
Schematic representation of various analytes used in liquid biopsy for varied clinical applications.

Liquid biopsies analyze circulating biomarkers, including DNA, RNA, proteins, and other chemicals contained in physiological fluids like blood, saliva, urine, stool, and pleural fluid, as opposed to conventional tissue biopsies, which can be invasive and difficult to collect (Mino-Kenudson, 2016; Raez et al., 2023). This method allows for the detection of genetic mutations and other biomarkers linked to tumors, such as circulating tumor cells (CTCs), circulating tumor DNA (ctDNA), cell-free DNA or RNA (cfDNA/cfRNA), tumor-educated platelets (TEPs), circulating tumor-derived endothelial cells (CTECs), exosomes, and protein molecules for early cancer detection (Vacante et al., 2020; Zhou et al., 2022). Since they offer clinicians access to real-time information about a patient’s cancer status without requiring repeated intrusive procedures, liquid biopsies are particularly useful in tracking therapy response and disease progression (Sivapalan et al., 2023) (Figure 1). Apart from capturing tumor heterogeneity, a liquid biopsy can facilitate recording broad temporal molecular footprints of the tumor, along with its changes throughout the disease progression to direct better clinical decisions (Lone et al., 2022; Shegekar et al., 2023) (Figure 1). The next section of this article discusses various components of body fluids that can be used as tools for liquid biopsy and are reported to predict disease states, survival outcomes, or treatment outcomes in various gastrointestinal cancers.

## 3 Circulating tumor cells

**FIGURE 2:**
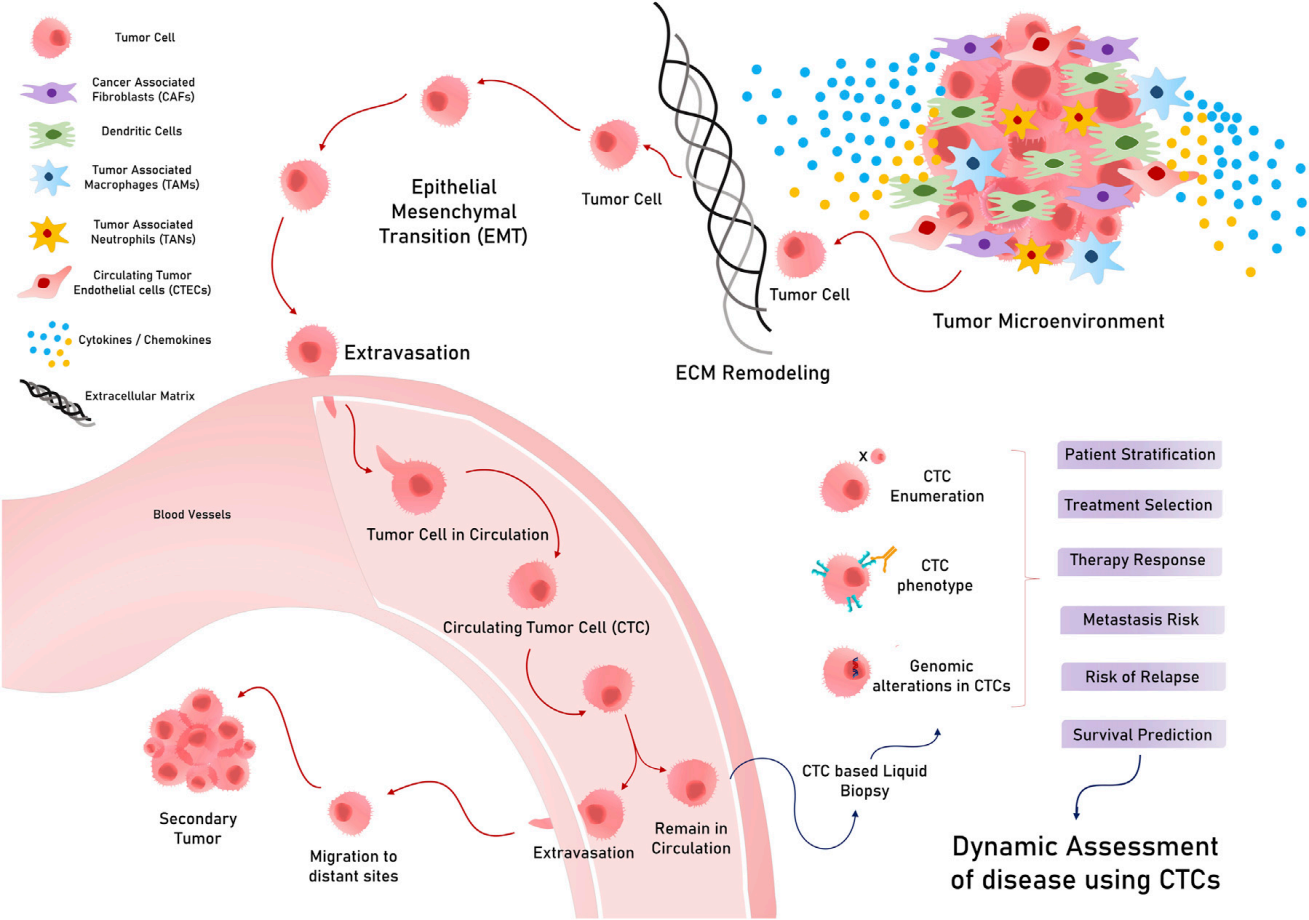
Circulating tumor cells (CTCs) in circulation and their various applications in cancer.

Circulating tumor cells are tumorous cells that exuviate from primary or metastatic tumors and extravasate to enter and stay in the blood and lymph circulation. These rare cells travel throughout circulation to eventually colonize a distant organ and form secondary tumors (metastasis) (Chauhan et al., 2021) (Figure 2). They exhibit considerable heterogeneity in terms of size, shape, and surface characteristics. A typical CTC is slightly larger than a white blood cell and can range from 10 to 30 um in diameter. These less frequently found cells (ranging from a few to up to hundred cells per ml of peripheral blood) can exist either as single cells or as clusters with stromal cells, platelets, or macrophages (Chen et al., 2022). Solid tumors of epithelial origin (breast, colon, prostate, etc.) usually generate CTCs, mostly expressing cytokeratins (CKs), along with epithelial cell adhesion molecules (EpCAMs). However, CTCs exhibit considerable diversity in terms of surface markers and cell types, which hinders the identification and isolation of clinically useful CTCs (Petrik et al., 2022).

CTCs, which are conventionally associated with the metastatic potential of any tumor, have potential applications in early diagnosis and screening. While considering CTCs for the early diagnosis and screening of GI tumors, it is important to consider the stage at which they manifest in a tumor (Yang C. et al., 2019). Early tumor lesions that can be detected using imaging methods, such as MRI/PET or CT scans, have approximately 109 cells and, hence, may start spreading disseminated tumor cells (DTCs) early, much before a metastatic tumor is diagnosed (Heiss et al., 1995; Friberg and Nystrom, 2015). In this regard, a study on various asymptomatic but high-risk individuals with a family history of any cancer type reported that 50% of them were positive for CTCs, while 20% developed early tumor lesions when followed up (Ried et al., 2017). Despite the theoretical and experimental rationale for considering CTCs for early diagnosis and screening, their extremely low occurrence in peripheral blood, and less sensitive technical methods to isolate them, CTC examination as a screening tool for early diagnosis in standard treatment has not been achieved, possibly because of technological and logistical limitations (Batool et al., 2023). Nevertheless, CTC detection in asymptomatic patients and patients with a family history of cancer has shown promising results in medium cohort studies, indicating its future prospects for use in screening strategies (Castro et al., 2018). However, CTCs have promising clinical applications, such as prognostic classification, metastatic monitoring, and prediction of response to therapies (Nikanjam et al., 2022) (Figure 2).

### 3.1 CTCs and patient groups

CTCs can also be utilized for patient stratification to provide beneficial treatment and enhance survival outcomes. In the case of CRC, CTCs are strongly associated with the stage of the disease, with more CTCs detected in advanced stages than in less advanced stages (Sastre et al., 2008; Bork et al., 2015). Notably, a study reported better diagnostic sensitivities of CTCs for early-stage CRC than serum biochemical markers, such as carcinoembryonic antigen (CEA) or CA19-9 (Yu et al., 2020). However, the CTC detection rate was not significantly associated with other clinicopathological features such as gender, location of the primary tumor (CEA), and lactate dehydrogenase (LDH) levels. However, its efficiency in detecting advanced-stage CRC remains unclear. A recent study reported that vimentin + CTCs could be used to better diagnose stage III/IV CRC (Cao et al., 2023). Moreover, research into the clinical utility of qualitative gene mutations in DNA isolated from CTCs, instead of their enumeration, has provided interesting insights. We already know that CRC-associated gene mutations with known clinical utilities, such as KRAS, APC, TP53, ERBB1/2, and FBXW7, may be evaluated in the CTC genome to predict accurate treatment options and interventions (Bidard et al., 2019) (Table 1). For instance, mutations in oncogene epithelial cell transforming sequence 2 (ECT2) were strongly associated with advanced CRCs, even more than serum CEA levels (Wang H. et al., 2019), supporting its usefulness in the prognosis of CRC (Chen et al., 2017) (Table 1). Furthermore, the expression of genes such as hTERT and MAGEA1-6 is more strongly associated with stage T3/T4 than stage T1/T2 in CRC (Kim et al., 2017).

**TABLE 1:** Representative studies relating circulating tumor cell (CTC) detection for various clinical applications in gastrointestinal (GI) tract cancers.

CTCs for patient classification in GI tract cancers					
Study	GIC type	Feature	Platform used	Level (quantitative or qualitative)	Summary
Sastre et al. (2008)	CRC	Tumor stage (I–IV)	CellSearch system	>2 CTC per 7.5 mL blood	Only tumor stages correlated with CTC detection rates even when >2 or >3 CTCs were considered positive CTC cut-off
Bork et al. (2015)	CRC	Tumor stage (I–III)	CellSearch system	>1 CTC per 7.5 mL blood	Proportion of patients with positive CTC count increase as the stage increases
Bork et al. (2015)	CRC	Tumor stage (IV)	CellSearch system	>2/>3 CTC per 7.5 mL of blood	Metastatic patients had higher CTC detection rates
Kang et al. (2017)	GC	Healthy vs GC group	Lab-on-a-disk method	2 CTC per 7.5 mL of blood	GC patients could be distinguished from normal patients with a sensitivity and specificity of 85.3% and 90.3%, respectively
Kang et al. (2017)	GC	Early-stage GC	Lab-on-a-disk method	A scorable CTC count of at least 1 CTC per 7.5 mL of blood	80% early-stage GC (T1,N0) had a scorable CTC count
Luo et al. (2018a)	HCC	Invasive HCC/Barcelona Clinic Liver Cancer (BCLC)	CanPatrol CTC analysis system	CTCs subgrouped as epithelial, mesenchymal, and mixed phenotypes	Mesenchymal and mixed CTC phenotypes associated with invasive or BCLC stages
Chen et al. (2019)	HCC	Tumor stage	FAST disk microfluidic system	2 CTCs per 7.5 mL of blood	No strong association was found between the stages and CTC count
Chen et al. (2019)	HCC	Metastatic HCC	CanPatrol CTC analysis system	3/>3 CTCs per 7.5 mL of blood	High CTC count can predict the risk of EHM in HCC patients
Wei et al. (2019)	PDAC	PDAC patients vs. healthy individuals	Microfluidic assay	2/>2 vimentin + CTC per 4 mL of blood	76% of PDAC patients had vimentin + CTCs
Song et al. (2021)	PDAC	Early-stage PDACs	Microfabricated porous filter-based CTC enrichment and flow cytometric enumeration after immunostaining	EpCAM + CTCs enumerated	EpCAM + CTCs and plectin-1 + CTCs evaluated in portal and peripheral blood in resectable PDAC patients may be used as potential diagnostic and prognostic markers
				Plectin-1 + CTCs enumerated	

CTCs for prognostic and therapy response applications in GI tract cancers					
Study	GIC type	Platform used	Scoring characteristic	Enumeration window	Comment
Yokobori et al. (2013)	CRC	autoMACS Cell sorting and immunocytochemistry	Evaluation of PLS3-expressing CTCs	Pre-operative peripheral blood of CRC patients	PLS3-positive CTCs represented as independent prognostic markers
Lee et al. (2015)	Metastatic GC	Anti-EpCAM antibody-coated magnetic particles using the CTC Profiler (Veridex)	>5 CTCs per 5 mL of blood	During the start of various chemotherapy cycles	Positive CTC score associated with unfavorable PFS and OS
Li et al. (2016)	Advanced GC	CellSearch	>3 CTCs per 7.5 mL of blood	6 weeks after chemotherapy	Predicted shorter PFS and OS
Liu et al. (2017)	Advanced GC	CELLection™ Epithelial Enrichment kit	>2 CTCs per 2 mL of blood	After the 1st cycle of chemotherapy	Predicted poor PFS and OS
Mu et al. (2014)	Early HCC	Negative enrichment of CTCs and Captor^TM^ system	16 CTCs per 7.5 mL of blood	Pre- and post-operative enumeration was done	CTC count decreased after successful surgery
Fang et al. (2014)	Intermediate HCC	Enrichment of CTCs by anti-EpCAM antibody and enumeration by fluorescent imaging	>1 CTC per 7.5 mL of blood	Pre- and post-TACE status of CTCs was evaluated in all patients	CTC count had no significant difference between patients receiving TACE or without receiving TACE
Rau et al. (2020)	Progressive HCC	Enrichment of CTCs followed by immunostaining and counting	50 CTCs per ml of blood represented progressive disease	CTC evaluated for both advanced and metastatic HCC	High CTC count represented a progressive HCC disease
Zhou et al. (2020)	Resectable HCC	CellSearch system	>1 CTC per 7.5 mL of blood	CTCs evaluated once after surgery	High CTC group reported high mVI counts and, therefore, more chances of recurrence
Su et al. (2022)	HCC	CytoSorter™	>2 CTCs per 2 mL of blood	Start and end of triple therapy, i.e., anti-PD-L1 therapy, anti-angiogenic therapy, and intensity modulated radiotherapy (IMRT)	Lesser score of PD-L1+ CTC at baseline was predictive of higher ORR and higher OS.
Poruk et al. (2017)	Resectable PDACs	Isolation by the size of epithelial tumor (ISET), followed by immunofluorescence	Various TIC surface phenotypes on CTCs were evaluated	CTCs evaluated in PDAC patients undergoing surgery	Patients positive for one or more CTC-TIC phenotypes act as independent factors for reduced OS) and DFS
Court et al. (2018)	Resectable PDACs	NanoVelcro Microfluidic System	3/>3 CTCs per 5 mL of blood	CTCs evaluated in PDAC patients undergoing surgery	High CTC count pre-operatively predicts chances of occult metastasis
Zhu et al. (2021a)	Resectable PDACs	Immunomagnetic separation and fluorescent cell counts	Various CTC phenotypes grouped according to desired fluorescent tags	CTCs evaluated in PDAC patients undergoing surgery	Patients having >50% CTCs as KLF8+/vimentin+ have reduced TTR and OS
Park et al. (2021)	Resectable PDACs	CD-PRIME platform	Various CD144/EpCAM/CK CTC surface phenotypes	CTCs evaluated in PDAC patients undergoing surgery	CTC positivity defined by EpCAM/CK+ CD44^−^ was associated with higher chances of early or frequent recurrence and systemic recurrence

CRC, colorectal cancer; GC, gastric cancer; HCC, hepatocellular carcinoma; PDAC, pancreatic ductal adenocarcinoma; CTCs, circulating tumor cells; EHM, extrahepatic metastasis; FAST, fluid-assisted separation technology; IMRT, intensity modulated radiotherapy; TACE, transarterial chemoembolization; OS, overall survival; PFS, progression-free survival; DFS, disease-free survival.

In GC, two or more CTCs in the blood could differentiate GC patients from healthy controls, whereas a scorable CTC level could predict a high proportion of early-stage GC (T1,N0) patients (Kang et al., 2017), indicating its potential role in distinguishing patients with early-stage GC for screening and diagnosis. Interestingly, in the case of hepatocellular carcinoma (HCC), various reports indicate conflicting findings pertaining to stage-dependent associations (Chen et al., 2019; Ha et al., 2019). Few studies have reported a positive CTC count that is not associated with any clinical features, including tumor stage (Chen et al., 2019), while others have indicated that CTC levels are associated with hepatitis B, the co-existence of satellite nodules, and alanine aminotransferase levels (Ha et al., 2019). However, an interesting study that sub-grouped CTCs into epithelial/mesenchymal or mixed surface biology indicated that mesenchymal and mixed CTCs are strongly associated with a higher clinical stage and stronger invasive properties in HCC (Luo C.-L. et al., 2018). In addition, CTCs also provide valuable information for patients undergoing surgery. A post-operative CTC detection of >3 CTC per 7.5 mL of blood can distinguish patients with a risk of extrahepatic metastasis (EHM) in HCC (Sun et al., 2020). Similar to HCC, where the advanced disease stage has a mesenchymal CTC phenotype, pancreatic ductal adenocarcinoma (PDAC) also has CTCs that have the mesenchymal surface marker vimentin on their surface, which could differentiate PDAC patients from healthy patients (about 76% were positive for vimentin + CTCs) (Wei et al., 2019; Arnoletti et al., 2022). Another qualitative evaluation of plectin-1 + CTCs and EpCAMs + CTCs in the portal and peripheral blood could identify patients with early-stage resectable PDAC, which can potentially be used as an early diagnostic biomarker (Song et al., 2021). Additionally, in pancreatic cancer cases lined for surgery, pre-operative vimentin + CTC evaluation was associated with a more advanced stage of disease and metastasis (Table 1) (Wei et al., 2019). Likewise, CTC evaluation in several GI tract cancer cases can serve as a tool for patient classification to direct informed therapy prescriptions (Figure 2).

### 3.2 Prognosis and treatment response prediction using CTCs

**FIGURE 3:**
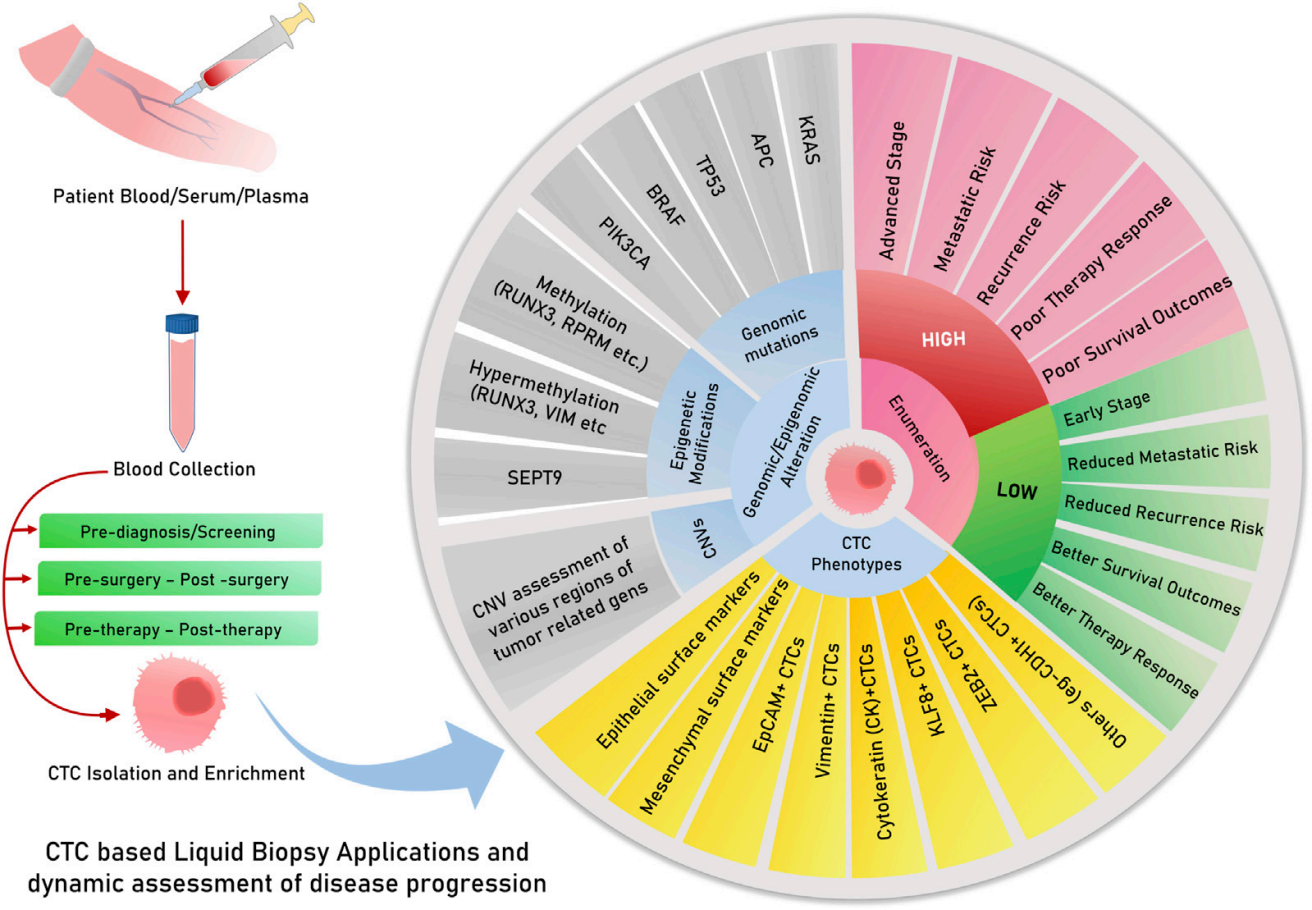
Circulating tumor DNA/cfDNA have multiple features and modifications, which are explored and tested for clinical decisions about risk, relapse, survival, and therapy responses. cfDNA or ctDNA is useful for dynamic monitoring, therapy responses, and risk assessment of relapse. Possibilities for the risk of assessment of CTCs for prognostic and other clinical applications.

CTC evaluation in cancers is strongly associated with the disease stage, along with their utility in predicting patient survival and therapy responses, which can be exploited for therapy monitoring applications and predicting outcomes in patients. In CRC patients, a study indicated that high CTC levels in the blood are significantly associated with inferior progression-free survival (PFS) and reduced overall survival (OS) (Cohen et al., 2009) (Figure 3). Moreover, a positive CTC score was closely correlated with the tumor stage in both pre- and post-operative scenarios, and interestingly, only post-operative CTC levels were positively associated with relapse-free survival (RFS) (Yang et al., 2018), indicating the clinical utility of post-operative CTC enumeration. For non-metastatic CRC (stages I–III), primary tumor features were not significantly associated with CTC detection, but a high CTC count during chemotherapy was also associated with unfavorable PFS (Wang et al., 2019b). It is already known that CTC levels are strongly associated with the TNM stage, lymphatic invasion, CEA levels, and distant metastasis (Sun et al., 2020). Several studies have, therefore, concluded that CTC scores are independent prognostic factors for predicting PFS and OS in CRC (Zhang et al., 2017; Bidard et al., 2019; Dizdar et al., 2019; Nicolazzo et al., 2019), whereas others have noted them as insignificant in some special cases, such as during pulmonary metastasectomy of metastatic CRC (Le et al., 2018). The genomic profiles of CTCs can also serve as prognostic indicators. Some genes found in CTCs play a role in the prognostic classification of CRC, including COX-2 (Cai et al., 2019), LGR-5 (Wang et al., 2018), and AKT-2, wherein positive AKT-2 expression may predict shorter median survival, PFS, and OS (Welinder et al., 2015) (Table 1). Moreover, the overexpression of plastin 3 (PLS3) on the CTC surface is an independent predictor of CRC patient prognosis, strongly associated with the DukeB and DukeC stages of CRC (Yokobori et al., 2013).

In GC, a high CTC count has been associated with reduced overall survival rates in multiple studies and meta-analyses (Huang et al., 2015). Studies have also reported that an unfavorable or high CTC count after 6 weeks of chemotherapy predicts reduced PFS and OS in addition to the objective response rate (ORR) in advanced gastric cancers (Li et al., 2016). Another study employing the enrichment of the epithelial marker EpCAM, followed by immunostaining and enumeration, could predict a positive CTC count to be associated with poor PFS and OS, even in patients undergoing their first cycle of chemotherapy (Liu et al., 2017). However, in metastatic GCs, a positive CTC count was found to predict poor PFS and OS in patients undergoing various chemotherapy cycles (Lee et al., 2015) (Table 1; Figure 3). A recent meta-analysis on gastric cancer, however, identified the immunofluorescent detection of CTCs to produce a significant association between CTC-positive counts and overall survival compared to molecular detection methods (Li Q. et al., 2022). This highlights a possible reason for inconsistent CTC enumeration results due to a technical bias caused by the platform/methodology followed by various studies across the globe.

When considering HCC, CTC scores can predict responses to surgery and therapy. For instance, pre- and post-operative CTC detection by negative enrichment and the Captor™ system could efficiently report a reduction in the CTC count after successful hepatectomy (Mu et al., 2014). Studies have also reported that a positive CTC score is associated with lower survival and recurrence rates (Ha et al., 2019). More than 3 CTCs per 4 mL of blood are associated with shorter recurrence-free survival and worse overall survival in pancreatic cancer patients (Chen et al., 2019; Wei et al., 2019). In another study, a positive CTC count was associated with lower survival and a higher chance of relapse among HCC patients with low levels of alpha-fetoprotein and a co-occurrence of cirrhosis (Ha et al., 2019). CTC scoring can also be utilized to predict progressive disease (PD) in cases of HCC. For instance, a study reported that a higher CTC count is predictive of PD and that a lower CTC count could represent a stable disease (SD) or partial response (PR) in HCC. This study reported 50 CTCs per ml blood for PD and approximately 15 CTCs per ml of blood for SD and PR (Rau et al., 2020). Another risk factor assessed post-surgery is the evaluation of microvascular invasion, which can enhance the chances of recurrence in patients with HCC. Zhou et al. (2020) found that a positive CTC score is strongly associated with enhanced mVI counts, thereby increasing the chances of relapse/recurrence. The authors of this study are of the informed opinion that the CTC-positive group must have a surgical margin >1 cm from the tumor to minimize the chances of relapse and increase the overall survival of HCC patients (Zhou et al., 2020). Furthermore, intermediate-stage HCC patients who received transarterial chemoembolization (TACE) did not have significantly lower CTCs than patients who did not receive TACE, elucidating the lesser role of CTC enumeration in this patient subgroup (Fang et al., 2014) (Table 1).

Similar studies and meta-analyses in PDAC patients have found CTC detection to be useful in predicting lower overall survival and reduced progression-free survival (Han et al., 2014; Martini et al., 2019; Wang et al., 2020). Many studies in resectable PDAC patients have reported that pre-operative CTCs are associated with lower survival and chances of relapse (Poruk et al., 2017; Zhu B. et al., 2021; Hugenschmidt et al., 2021; Park et al., 2021). More recently, in a study, time to recurrence (TTR) was found to be reduced in patients with >50% CTCs as KLF8+/vimentin+, along with the perineural invasion status (Zhu P. et al., 2021). A positive CTC evaluation (EpCAM/CK+ and CD44^−^) in patients with resectable PDAC can also predict the chances of early, frequent, and systemic recurrence (Park et al., 2021). CTC phenotypes representing tumor-initiating markers comprised one or more markers, such as aldehyde dehydrogenase (ALDH), CD44, or CD133, indicating a tumor-initiating cell (TIC) phenotype, which could act as an independent factor for reduced OS and disease-free survival (DFS), as reported in a recent study (Poruk et al., 2017). Similarly, a few studies reported that zero or less than one CTC with EpCAM and Plectin-1 mesenchymal markers was associated with longer overall survival and can be used as a prognostic marker (Song et al., 2021). The milestone CLUSTER study was designed to evaluate CTC dynamics and found that pre-operative CTC levels were strongly associated with early recurrence in patients undergoing neoadjuvant therapy (NAT) or initial resection surgery. In the neoadjuvant group, undetectable CTCs were correlated with longer overall survival, reiterating the popular fact that the presence of CTCs is indicative of poor survival (Gemenetzis et al., 2018). However, pre-operative CTC levels could also be used to identify patients with a high chance of occult metastasis from others, as suggested by a study employing the NanoVelcro microfluidic system for CTC evaluation (Court et al., 2018). More recently, a new circulating stromal cell named cancer-associated macrophage-like cell (CAML) has been identified; a higher count of which is predictive of advanced stage and reduced progression-free survival, along with the identification of more aggressive forms of pancreatic cancer (PC) (Gardner et al., 2021) (Table 1).

Dynamic evaluation of CTC status in patients during the course of treatment would prove useful to clinically judge the response to therapies and decide the change of course if required. CTCs were observed to decrease in peripheral blood after patients received cryotherapy for liver metastasis, along with a reduction in other serum biomarkers such as CEA, CK18/19, and EpCAM (Shi et al., 2016) (Figure 3). Another study evaluating patients receiving anti-PD-L1 treatment reported high PD-1/PD-L1 expression in CTCs as a biomarker for screening patients for this therapy (Yue et al., 2018). For patients receiving the popular FOLFOX and bevacizumab treatment for mCRC, a decreasing CTC count and VEGFR positivity in CTC corresponded to better treatment responses, as reported in a study (Delgado-Ureña et al., 2018) (Table 1). Therefore, the above studies point to the important and actionable roles of CTCs and CTC-phenotypes in capturing tumor evolution during the course of therapy for the effective monitoring of treatment response and yielding better outcomes (Figure 3).

## 4 Tumor-educated platelets

Platelets are abundant anucleated cell types found circulating in body fluids like blood and lymph, originating from megakaryocytes. They play a well-known role in maintaining homeostasis and actively participating in the wound-healing process of thrombosis (van der Meijden and Heemskerk, 2019). Tumor-educated platelets are platelets that pick up tumor-associated biomolecules and vesicles while interacting with tumor cells (Nilsson et al., 2011) in the tumor microenvironment (TME). Platelets have a strong association with wound healing and inflammation, which are also frequent events occurring in the tumor progression pathway, indicating the formidable role of this cellular species in cancer prognosis. The first report of platelets being vehicles of biomolecular signals from tumors was reported when a study discovered their ability to attach to tumor RNA-containing vesicles (Nilsson et al., 2011). Tumor cells can directly initiate the development of circulating tumor-educated platelets by interacting with them or through various factors such as RNA/vesicles/proteins released from them to educate them to eventually emerge as TEPs (Plantureux et al., 2018). Many studies lay out the process of how tumor cells activate circulating platelets and initiate the formation of TEPs (D’Ambrosi et al., 2021; Xu et al., 2018). Across cancer types, studies stated the valuable role of TEPs in carrying tumor mutational signatures as RNA profiles and indicated their potential role fit to be used for pan-cancer diagnostic utilities (Best et al., 2015). Many studies in the scientific domain report mRNA profile changes in TEPs in various cancer types, such as non-small cell lung cancer (NSCLC) (Luo L. et al., 2018; Xue et al., 2018; Dong et al., 2021), breast cancer (Mendoza-Almanza et al., 2020), liver cancer (Best and Wurdinger, 2020; Waqar et al., 2021), and glioma (Campanella et al., 2020). Some studies have also investigated and found the role of medium-sized extracellular vesicles (EVs) (mEVs) exuviated by platelets activated by thrombins to carry prognostic clues (Contursi et al., 2023). TEPs and their role in the clinical utilities of CRC are still an under-researched area; however, their potential role in prognosis and treatment monitoring is not disputed. TEPs can, singularly or along with other diagnostic biomolecules such as CTCs, CAFs, or ctDNA, be used for clinical utilities such as early diagnosis, screening, treatment monitoring, and predicting prognosis in cancer patients.

TEPs have several clinical utilities in various cancer types apart from gastrointestinal cancers, including breast cancers (Best et al., 2015; Alimirzaie et al., 2019; Yao et al., 2019; Mendoza-Almanza et al., 2020), nasopharyngeal cancers (Wang et al., 2019c), prostate cancers (Tjon-Kon-Fat et al., 2018; Boerrigter et al., 2020), lung cancers (Sheng et al., 2018; Xing et al., 2019), glioblastoma (Campanella et al., 2020; Sol et al., 2020; Meng et al., 2021), and ovarian cancer (Mysona et al., 2019; El-Arabey et al., 2020). Most studies evaluating the role of TEPs have qualitatively reported the RNA profiles of TEPs, whereas the protein profiles of TEPs still remain considerably unexplored. For instance, a TEP RNA profiling study reported that CRC-associated signatures in RNA profiles could efficiently differentiate between CRCs and other non-cancerous colon-related diseases like ulcerative colitis (UC), Crohn’s disease, and non-cancerous polyps (Xu et al., 2022). Similarly, a study of TEP proteins concluded a strong association between CRC disease state and higher levels of platelet-derived growth factor (PDGF), platelet factor-4 (PF4), and vegetative epithelial growth factor (VEGF) in TEPs employing ELISA (Peterson et al., 2012). In addition to proteins, in CRC, the presence of a higher TIMP1 mRNA level was also reported, which could efficiently differentiate the diseased group from the healthy group, indicating its role in diagnosis (Yang L. et al., 2019). Given the biological role of PDGF and TIMP1 in angiogenesis and metastasis, a more inclusive study might indicate their role in differentiating metastatic/advanced CRC as opposed to early-stage CRC. Non-coding RNAs associated with TEPs have also been understood to have utilities in the clinical management of CRC. A study could identify high expression of four long non-coding RNAs (lncRNAs), namely, LNCAROD, TSPOAP-AS1, LINC00534, and another SNHG20, in patient serum and tumor-educated platelets isolated from CRC patients. In this, two lncRNAs, namely, LNCAROD and TSPOAP-AS1, were found to have strong associations with the primary tumor location and stage of the disease (Ye et al., 2022). Very recently, another study discovered that significant levels of the lncRNA named colon cancer-associated transcript-1 (CCAT1), usually associated with tumor TEPs, were highly expressed in the circulation of CRC patients (Tabaeian et al., 2024). Futuristic studies investigating mEVs, which were found to have distinct sizes and proteomic profiles compared to healthy individuals (Contursi et al., 2023), indicating their pro-metastatic roles, could be explored further along with other biomarkers to enhance their diagnostic and prognostic efficacies.

In pre-clinical models, pancreatic cancer cells are reported to stimulate the aggregation of platelets, a phenomenon known as tumor cell-induced platelet (TCIP) aggregation, which is followed by thrombosis; this can enhance disease progression by promoting intra-tumor communications (Mai and Inkielewicz-Stepniak, 2021). Moreover, TEPs and their role in defining the clinical characteristics of pancreatic cancers have not been well researched. However, many studies have underlined the clinical utilities of various platelet features in the range of PC characteristics like a higher mean platelet volume (Yin et al., 2018), predictive of liver-metastatic PC, and advanced stages (stages III–IV), while a decreased MPV was associated with poor prognosis and early-stage patient groups (Yagyu et al., 2021). TEPs and their RNA profiles could differentiate between KRAS wildtype and KRAS-deficient pancreatic tumors (Best et al., 2015). Proteome profiles of platelets could also differentiate between early stages (stages I–II) of the head of pancreas cancer and those of age and sex-matched healthy subjects (Sabrkhany et al., 2017). A bioinformatics investigation also revealed a possible role of TEP-associated mRNA RSL24D1 in being associated with an early pancreatic tumor disease stage compared to their healthy counterparts. New research deliberations are required in this area to bring stable biomarkers fit to be used in clinical settings and applications. As pancreatic cancer patients are at an increased risk of venous thrombosis, another possible research interest would be to investigate the association between platelets and thromboembolism-associated factors in patient prognosis as an earlier study on a pan-cancer cohort demonstrated increased levels of cancer-associated tumor thromboembolism (VTE)-associated factors such as ADAMTS-13 and VWF in patients having worse survival rates (Obermeier et al., 2019).

Platelets play an important physiological role in the liver, and therefore, in hepatic diseases such as hepatocellular carcinomas, their dysregulation is expectedly observed (Lambert, 2016). In HCC, high platelet counts were long associated with shorter survival (Schrecker et al., 2022) and may also indicate chances of early recurrence if considered along with CTCs (Lu et al., 2023). MicroRNAs isolated from TEPs also carry many cues of HCC-specific upregulations, such as enriched RhoA, SPINK1, and CTNNB1, along with a considerable upregulation of SERPIND1, IFITM3, and CD41^+^ levels, to demarcate HCC and cirrhosis patients (Waqar et al., 2021). Another study reported the high expression of TGF-ß, NF-KB, and VEGF in mRNA isolated from TEPs in HCC patients, indicating an advanced stage, whereas a reduced level of AKT and PIK3 could represent early-stage HCC (Asghar et al., 2020). Studies investigating microRNAs in TEPs isolated from HCC patients when computationally analyzed could identify a differentially expressed microRNA pair, i.e., miR-1293 and miR-495-3p in HCC patients, playing pathological and diagnostic roles (Zhu B. et al., 2021). These diverse utilities of TEPs underline more such investigations in larger cohorts for designing more precise prognostic models.

## 5 Circulating cell-free DNA

Circulating cfDNAs are DNA fragments in blood/plasma circulation (Mandel and Metais, 1948), mostly derived from healthy leukocytes and stromal cells, and found to be enriched in cancer patients (Leon et al., 1977). Their applicability in tumor diagnosis and prognosis was not recognized until studies indicated that a considerable proportion of cfDNA originates from tumors and can have tumor-associated footprints. In 1994, a remarkable study identified tumor-associated KRAS and NRAS gene mutations in cfDNA, which sparked further curiosity to unravel the diagnostic and prognostic purposes of cfDNA in various tumors (Caldas et al., 1994). In cancer patient serum and plasma, cfDNA is found at higher levels, mainly due to cellular apoptosis or necrosis of stromal and immune cells in tumors (Stejskal et al., 2023). Regarding patient diagnosis and classification, earlier studies indicated that cfDNA levels in CRC patients were enriched both in plasma and stool, paving the way for its potential use in less-invasive CRC diagnosis (Sidransky et al., 1992; Harlé, 2020). However, cfDNA elevation is also indicative of other diseased states relating to inflammatory and other physical stress conditions (Hummel et al., 2018). Therefore, its use, along with other minimally invasive markers such as tumor-specific gene mutations and their methylation status, has yielded greater prognostic and diagnostic specificity (Table 2).

**TABLE 2:** Representative studies relating cfDNA for clinical applications in gastrointestinal tract cancers.

Study	Cancer type	Feature	Platform used	Summary
Hamfjord et al. (2019)	CRC	CfDNA quantification	ddPCR	High pretreatment cfDNA levels represented reduced DFS
Wu et al. (2021)	CRC	Methylation biomarkers in cfDNA	Targeted DNA methylation sequencing	Early-stage CRC and advanced adenomas (AAs) could be diagnosed by these 11 DNA methylation markers in cfDNA
Wu et al. (2022)	CRC	cfDNA quantification	qRT-PCR	Lowered cfDNA levels after chemotherapy
				Represented better survival
Walker et al. (2022)	CRC	Hydroxymethylcytosine-based classifier in cfDNA	Capturing 5hmC regions in cfDNA and sequencing on the Illumina platform	The classifier could detect early-stage I CRC and healthy patients with satisfactory sensitivity, which increased when other cfDNA parameters such as size and abundance were considered
Sai et al. (2007), Zhang et al. (2019a)	GC	cfDNA	qPCR	Elevated plasma cfDNA concentration and integrity were found in GC patients contrasting healthy individuals
Chen et al. (2023)	GC	CSF-cfDNA	Next-generation sequencing	Levels of cfDNA in CSF could indicate the presence of metastasis
Tokuhisa et al. (2007)	HCC	cfDNA	Real-time PCR	CfDNA levels were significantly associated with non-HCV-infected HCC patients and less with HCV-infected HCC patients
Yang et al. (2011)	HCC	cfDNA	Real-time fluorescent qPCR	hTERT DNA in cfDNA was more elevated in HCC patients than in HBV-infected HCC patients
Zheng et al. (2021a)	HCC	cfDNA	Circulating single-molecule amplification and resequencing technology (cSMART)-based method (SIM)	HBV-associated integrational hotspots to identify and segregate HCC patients with HBV-associated risks and progression
Kim et al. (2023)	HCC	cfDNA	Sensitive PCR-based method known as methylation sensitive high-resolution analysis (MS-HRM)	This methylation signature panel with RNF135 and LDHB could more accurately detect HCC patients from at-risk and healthy individuals
Zitt et al. (2008)	CRC	cfDNA	RT-PCR	Reduced post-CRT, cfDNA level represented a better response to CRT
Li et al. (2017a)	CRC	cfDNA	Copy number variation assessment	High CNVs associated with shorter survival
Zhong et al. (2020)	CRC	cfDNA	q-PCR	Post-surgical higher cfDNA concentration was associated with poor PFS

CRC, colorectal cancer; GC, gastric cancer; HCC, hepatocellular carcinoma; PDAC, pancreatic ductal adenocarcinoma; CTCs, circulating tumor cells; EHM, extrahepatic metastasis; CNV, copy number variation; OS, overall survival; PFS, progression-free survival; DFS, disease-free survival.

Tumor-specific gene mutations arise from necrotic and apoptotic tumor cells, which release their fragmented DNA in circulation, giving rise to a separate subgroup within cfDNA known as ctDNA (Leung et al., 2016). This ctDNA is a vehicle for tumor-specific gene mutations, like KRAS (Sidransky et al., 1992), BRAF (Fu et al., 2021), TP53, APC, or PIK3CA (Jauhri et al., 2017). Therefore, the detection of these mutations in cfDNA would indicate disease states, chances of recurrence, or responses to therapies in a more focused manner. However, recent investigations showed that ctDNA detection rates are dependent on the quality and quantity of input cfDNA in DNA-sequencing platforms (Bettegowda et al., 2014). An interesting study indicated that 45% of CRC-specific mutations (KRAS) in tumor tissues could also be found in their plasma cfDNA in patients and not in healthy groups (Wang et al., 2004). The concordance of CRC mutations in both tumor tissue and plasma cfDNA was also studied, which underlined the concordance of 96% KRAS and 100% BRAF mutations (Thierry et al., 2014). Similarly, many studies reported higher levels of cfDNA in plasma, which was strongly correlated with the presence of CRC in healthy individuals (Pu et al., 2021; Wu et al., 2022); on the other hand, in another study, cfDNA was found to be enriched more in colon tumors (500 ng/mL) than in rectal tumors, which had plasma levels of approximately 250 ng/mL (Frattini et al., 2008) (Table 3).

**TABLE 3:** Studies relating CTC detection and various prognostic parameters in gastrointestinal cancers.

Study	GIC type	Platform used	Scoring characteristic	Enumeration window	Comment
Lee et al. (2015)	Metastatic GC	Anti-EpCAM antibody-coated magnetic particles using the CTC Profiler (Veridex)	>5 CTCs per 5 mL of blood	During the start of various chemotherapy cycles	Positive CTC score associated with unfavorable PFS and OS
Li et al. (2016)	Advanced GC	CellSearch system	>3 CTCs per 7.5 mL of blood	6 weeks after chemotherapy	Predicted shorter PFS and OS
Liu et al. (2017)	Advanced GC	CELLection™ Epithelial Enrichment kit	>2 CTCs per 2 mL of blood	After the 1st cycle of chemotherapy	Predicted poor PFS and OS
Mu et al. (2014)	Early HCC	Negative enrichment of CTCs and Captor^TM^ system	16 CTCs per 7.5 mL of blood	Pre- and post-operative enumeration was done	CTC count decreased after successful surgery
Fang et al. (2014)	Intermediate HCC	Enrichment of CTCs by anti-EpCAM antibody and enumeration by fluorescent imaging	>1 CTC per 7.5 mL of blood	Pre- and post-TACE status of CTCs was evaluated in all patients	CTC count had no significant difference between patients receiving TACE or without receiving TACE
Rau et al. (2020)	Progressive HCC	Enrichment of CTCs followed by immunostaining and counting	50 CTCs per ml of blood represented progressive disease	CTC evaluated for both advanced and metastatic HCC	High CTC count represented a progressive HCC disease
Zhou et al. (2020)	Resectable HCC	CellSearch system	>1 CTC per 7.5 mL of blood	CTCs evaluated once after surgery	High CTC group reported high mVI counts and, therefore, more chances of recurrence
Su et al. (2022)	HCC	CytoSorter™	>2 CTCs per 2 mL of blood	Start and end of triple therapy, i.e., anti PD-L1 therapy, anti-angiogenic therapy, and IMRT	Lesser score of PD-L1+ CTC at baseline was predictive of higher ORR and higher OS.
Poruk et al. (2017)	Resectable PDACs	Isolation by the size of epithelial tumor (ISET), followed by immunofluorescence	Various TIC surface phenotypes on CTCs were evaluated	CTCs evaluated in PDAC patients undergoing surgery	Patients positive for one or more CTC-TIC phenotypes act as independent factors for reduced OS and DFS
Court et al. (2018)	Resectable PDACs	NanoVelcro Microfluidic System	3/>3 CTCs per 5 mL of blood	CTCs evaluated in PDAC patients undergoing surgery	High CTC count pre-operatively predicts chances of occult metastasis
Zhu et al. (2021b)	Resectable PDACs	Immunomagnetic separation and fluorescent cell counts	Various CTC phenotypes grouped according to desired fluorescent tags	CTCs evaluated in PDAC patients undergoing surgery	Patients having >50% CTCs as KLF8+/vimentin+ have reduced TTR and OS.
Park et al. (2021)	Resectable PDACs	CD-PRIME platform	Various CD144/EpCAM/CK CTC surface phenotypes	CTCs evaluated in PDAC patients undergoing surgery	CTC positivity defined by EpCAM/CK+; CD44^−^ was associated with higher chances of early or frequent recurrence and systemic recurrence

CRC, colorectal cancer; GC, gastric cancer; HCC, hepatocellular carcinoma; PDAC, pancreatic ductal adenocarcinoma; CTCs, circulating tumor cells; EHM, extrahepatic metastasis; FAST, fluid-assisted separation technology; IMRT, intensity modulated radiotherapy; TACE, transarterial chemoembolization; OS, overall survival; PFS, progression-free survival; DFS, disease-free survival.

CTC-associated parameters such as cfDNA abundance and integrity (Pu et al., 2021; Wu et al., 2022) are also stage-dependent and could be used for monitoring disease progression. However, studies have failed to show a compounded cfDNA trend across studies; instead, cfDNA abundance and growth rates at endpoints of various clinical crossroads, such as post-therapy and pre-therapy, have found evidential backing in studies. Moreover, in a recent large-scale study of 16,347 patients in stages I–III of CRC, post-operative cfDNA levels did not affect ctDNA positivity, which clearly demonstrated the better usefulness of ctDNA in driving clinical decisions (Cohen et al., 2023).

Currently, DNA methylation markers in cfDNA are being researched for more accurate patient classification exercises. In this thread, a study found that an 11-DNA methylation marker model could effectively distinguish between early-stage CRC and advanced adenomas and can help guide early diagnosis (Wu et al., 2021). Another recent finding is the role of 5-hydroxyl cytosine modifications in cfDNA. In a study, 5-hydroxymethylcytosine regions were captured and sequenced to develop a 5hmC profile of CRC and GC patients. This classifier could diagnose early-stage I and late-stage IV diseases in healthy individuals (Walker et al., 2022). The sensitivity of this method increased as it included other cfDNA features, such as the size of fragments and abundance, along with these 5hmC profiles (Walker et al., 2022), indicating a novel role of 5hmC regions of cfDNA in early CRC and GC diagnosis.

In hepatocellular carcinoma patients, cfDNA levels are approximately 20 times more enriched than those in healthy individuals (Iizuka et al., 2006; Tokuhisa et al., 2007; Huang et al., 2012). Patients undergoing hepatectomy project a longer survival rate and lesser recurrence risk if cfDNA levels reduce after surgery (Tokuhisa et al., 2007). HCC patient subgroups include those with liver cirrhosis, chronic hepatitis, or HBV-infected groups. cfDNA can be used to quantitatively recognize these subtypes. For instance, hTERT DNA in cfDNA was found to be more enriched in non-HBV-infected HCC patients than in HBV-infected HCC patients (Yang et al., 2011) and HCV carrier HCC patients (Tokuhisa et al., 2007). HBV infections damage the host DNA and are responsible for HCC progression. A study identified HBV integration hotspots in the cfDNA of HCC patients (Zheng B. et al., 2021) to be potentially used for surveillance and HBV-related risk stratification in HCC patients. The methylation of cfDNA is also predictive of HCC, such as the methylation of the cfDNA-based assay with a sensitivity of 78.5% and a specificity of 89% that could distinguish the diseased group from the healthy group (Wang C. et al., 2021). Another cfDNA-based methylation signature panel with ring finger protein 135 (RNF135) along with lactate dehydrogenase B (LDHB) studied healthy individuals, individuals at risk, and HCC patients to positively diagnose 57% of HCC patients better than the A-fetoprotein analysis (Kim et al., 2023). Recent interest lies in developing a multi-omics assay that integrates the mutational and epigenomic status in cfDNA to predict HCC in a more precise method (Wang P. et al., 2022) (Table 3).

In pancreatic cancers, cfDNA features have several associations with the disease state and stage. Apart from elevated levels of cfDNA in PDAC patients (Bronkhorst et al., 2019), cfDNA fragment lengths are also found to be shortened in individuals with such diseases (Ranucci, 2019). For the precise detection of stages, more specific genomic signatures in cfDNA (ctDNA) are required. The most frequently mutated genes in PDAC are KRAS, SMAD4, CDKN2A, and TP53, among various other genomic markers that could be assessed for differentiating the diagnosis of early and metastatic PDAC cases. A study reported that genetic alterations in cfDNA could be more specifically found in metastatic PDAC in contrast to fewer such alterations in local PDAC (Bettegowda et al., 2014).

Regarding prognosis and therapy response, as a trend, various studies have reported a decrease in cfDNA levels after chemotherapy (Wu et al., 2022). Patients who had higher cfDNA levels before first-line chemotherapy (oxaliplatin-based) showed reduced survival in terms of DFS in metastatic CRC (Hamfjord et al., 2019). On the other hand, patients who underwent surgery and had enhanced levels of cfDNA experienced lower PFS (Zhong et al., 2020). Numerous studies also record that in patients receiving various therapies, those who registered a reduction in cfDNA levels compared to pre-therapy cfDNA levels represented a better survival and therapy response group (Leon et al., 1977; Sozzi et al., 2001; Sozzi et al., 2003). Patients who received chemoradiotherapy (CRT) were reported to either have increased cfDNA levels or decreased cfDNA levels post-CRT compared to their initial pre-CRT cfDNA levels. Those patients who recorded reduced cfDNA levels after CRT were found to better respond than those with consistently high cfDNA levels (Zitt et al., 2008). Copy number variations (CNVs) are yet another feature of cfDNA that could indicate tumor progression and provide survival predictions. A positive test for enhanced CNVs in several chromosomal regions and tumor-associated genes found in cfDNA is associated with shorter survival (Li J. et al., 2017). However, more large-scale studies are needed to make strong predictions for the early diagnosis and definitive prognosis of CRC.

In gastrointestinal cancers like CRC, plasma cfDNA is found to be elevated in multiple studies (Sai et al., 2007; Zhang H. et al., 2019), both in terms of its concentration and integrity. The majority of individuals with gastroesophageal cancers (GECs) present with an advanced stage at diagnosis or collapse due to disease recurrence even after the main tumor is surgically removed, underlining the fact that GECs have high death rates. Such disease progressions can be predicted using tumor-specific genetic alterations in cfDNA isolated from plasma (Rumiato et al., 2017; Koldby et al., 2019). As already stated, in CRC, the methylation status of specific tumor-associated markers has better prognostic efficacy, which has also been noted in gastric cancers. A recent study identified the elevated status of methylated genetic markers in RUNX3, P16, RASSF1A, and RPRM in gastric cancers, unlike in healthy individuals. The study also underlines the greater role of RNX3 and RPRM in detecting early-stage (I–II) GC (Saliminejad et al., 2020).

In HCC, cfDNA assessment during the administration of systemic therapies and follow-up can capture tumor heterogeneity and actionable mutations from tumors, direct therapy, and offer progress monitoring tools (Coto-Llerena et al., 2022). Many therapy-responsive gene mutations also exist, such as CTNB1 S33C (Lin C. et al., 2021), ARIDIA R727, AXIN1 (Higuera et al., 2022), NF1 (Lyu et al., 2022), and NRAS, which can be detected well in cfDNA/ctDNA and can, therefore, direct therapy decisions in HCC patients. Higher cfDNA levels, as expected, supported reduced survival rates (Iizuka et al., 2006) and a higher risk of recurrence with elevated chances of metastasis (Huang et al., 2012).

## 6 Circulating tumor DNA

As discussed in the above section, ctDNA is a new and promising biomarker for appropriate, directed treatment in precision oncology using liquid biopsy. Tumor DNA, as ctDNA, can be used to analyze various tumor-related features such as prognosis, therapy responses, or heterogeneous resistance in individual patients, which are typically overlooked by tumor biopsies. The dynamics of repeated tumor biopsies have reduced patient compliance (Li W. et al., 2022) and may not be necessary after the discovery of ctDNA in many cancer types. Equivalent to next-generation sequencing (NGS) whole-genome sequencing, cfDNA and ctDNA are the most invaluable and highlighted blood biomarkers in recent years due to their predictive and prognostic importance. The foremost challenge to detecting ctDNA in the plasma is the presence of low-quantity DNA with a half-life of approximately 2 h. In contrast, ctDNA is present in higher amounts in cancer patients than in healthy individuals. The proportion of ctDNA in total plasma cfDNA increases for advanced stages (10%) while remaining very low in early stages; the exosomal prion proteins were found to be overexpressed in CRC, which could be cancer (0.1%). Less invasive procedures for testing ctDNA may help in the screening/diagnosis of early tumors, but its strong roles have been more extensively researched for the disease monitoring and molecular assessment of patient recovery, responses, and chances of relapse during and after the respective treatments. Although several studies showcase that ctDNA detection is more helpful in the advanced stages of tumors in detecting the heterogeneity of disease, a large-scale longitudinal study reported that using methylation markers in ctDNA, some major gastrointestinal tumors can be diagnosed as early as 4 years before they are detected by current standard diagnostic procedures (Chen et al., 2020a).

### 6.1 ctDNA and patient stratification

Circulating tumor DNA can play a role in early screening. Few studies claim that ctDNA detection can help in the pre-diagnosis of cancer even before the symptoms appear. In a recent study, ctDNA methylation was detected in the plasma of 605 individuals with no symptoms, and among them, 191 patients within 4 years were detected with various GI cancers (colorectal, esophageal, stomach, and liver cancer) (Chen et al., 2020b). However, more such studies are needed for conclusive evidence for its use in screening and early diagnosis. In addition to this, ctDNA features can harbor clues for patient stratification for precision therapy decisions. Circulating tumor DNA with a CRC-specific mutation can also be an important parameter for diagnosing clinically relevant tumor features. In metastatic CRC, ctDNA with KRAS mutations could detect diseased groups with 99.2% specificity and 87.2% sensitivity (Bettegowda et al., 2014), whereas its sensitivity to detect stage I CRC was not satisfactory. The inclusion of a more diverse gene panel of 38 genes indicated enhanced diagnostic abilities in gastric cancer, as reported by a study (Varkalaite et al., 2021). The methylation status of ctDNA could also provide discriminatory capability to distinguish between early disease and healthy individuals. A study concluded that the presence of six methylation markers in ctDNA was enriched in about 78% of stage I–III CRC patients (Mo et al., 2023) and that the methylation status of the *SEPT9* gene in plasma is strongly capable of distinguishing GC patients from healthy individuals least invasively (Bergheim et al., 2018; Zhao et al., 2022) (Table 3).

In PC, a four-gene (*PXDN*, *ADAMTS1*, *LRFN5*, and *BNC1*) methylation status assessment in ctDNA could provide 90% specificity in the early detection of PCs (Ying et al., 2021). Later, an interesting study designed a sequencing data analysis tool/algorithm called methylated CpG tandem amplification and sequencing (MCTA-Seq), utilizing 153 gene methylation assessments in ctDNA, which could diagnose GCs in various stages and could also discriminate between chromosomal instability methylator phenotype (CIMP) and non-CIMP gastric cancers. This algorithm and gene panel could also differentiate between early GC from CRC or HCC samples (Ren et al., 2022). This method has been applied to various other gastrointestinal cancer types for its early diagnostic utilities in respective types such as CRC (Li et al., 2019) and HCC (Wen et al., 2015).

In gastric cancer, a separate sub-type, Epstein–Barr virus (EBV)-GC, is identified by the presence of EBV infection. This subtype can be diagnosed by qPCR methods, which detect EBV sequences in tissue DNA. However, this subtype can also be efficiently diagnosed using cfDNA, with a sensitivity of 71% and a specificity of 94%. In addition to detecting the EBV subtype, this method can also help in assessing tumor progression and therapy responses in EBV-positive patients (Shoda et al., 2017). In many cases, it has been observed that cfDNA/ctDNA integrity is reduced in various cancer types as they are reported to have many regions of ultrashort or jagged DNA. Quite recently, technologies and tools were innovated that could efficiently capture even these ultrashort or jagged DNA sequences in ctDNA. A sequencing tool called broad-range cell-free DNA-Seq (BRcfDNA-Seq) can capture these ultrashort cfDNA fragments isolated from saliva and could discriminate between gastric cancer patients and healthy individuals (Swarup et al., 2023). Few rare GC cases metastasize in the leptomeningeal region, giving rise to gastric cancer with a leptomeningeal metastasis (GCLM) subtype. Quantifying post-therapy cfDNA in the CSF of such cases provides various prognostic clues, such as this study (Chen et al., 2023), which reported that reduced cfDNA levels in the CSF indicated better PFS in GCLM patients. In such cases, identifying levels and genomic alterations in CSF ctDNA could hold promising potential to provide prognostic decisions and clinical assessment. Many ongoing clinical trials are also identifying and mapping methylomes in GC patients for potential use in the early diagnosis of GC (Han et al., 2023).

### 6.2 ctDNA for prognosis and therapy response

ctDNA assessment before and after treatments can also be used to predict therapy responses. A study found a difference in the ctDNA level in mCRC patients before and after the treatment with FOLFIRI, where patients were found positive for increased ctDNA levels after the first cycle of chemotherapy and showed shorter PFS and poor survival (Lyskjær et al., 2019). Studies have shown the importance of ctDNA detection in the treatment with standard chemotherapy drugs. Hence, it is suggested that ctDNA detection in the first week of FOLFIRI treatment in colorectal cancer patients could direct the progression of diseases, survival outcomes, and the indication for treatment failure in patients. KRAS and NRAS are commonly mutated genes in CRC and are also an indicator for the response to anti-EGFR-based targeted therapy using monoclonal antibodies like panitumumab and cetuximab. A study investigated the role of ctDNA in the early prediction of responses to these anti-EGFR therapies (Vidal et al., 2017). In addition to this, the presence of CRC-specific mutations such as KRAS, APC, and p53 in ctDNA is associated with a higher chance of metastasis/recurrence in patients not harboring these mutations (Wang et al., 2004).

**FIGURE 4:**
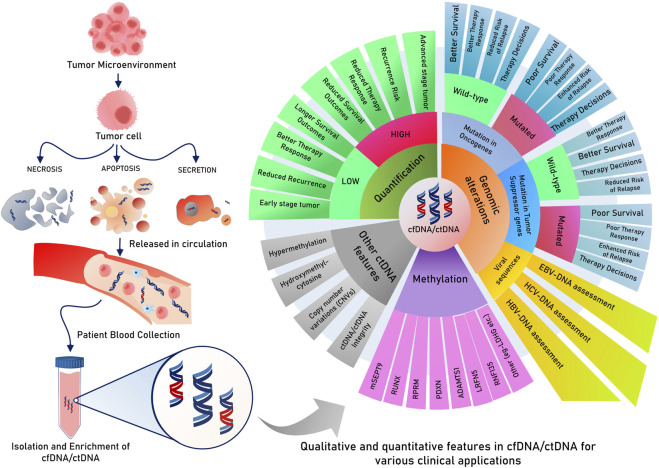
Various features of cfDNA/ctDNA in tumor-related clinical applications.

However, another recent study also underlined the relevance of scoring ctDNA positivity in cfDNA using a wide range of CRC-specific gene panels that can be used to predict recurrence risk in post-operative stage II/III patients and can guide the administration of adjuvant radiotherapy (ART). Here, the study reported post-operative ctDNA positivity with an enhanced risk of recurrence (Chen et al., 2021) and can help in the selection of high-risk patients for ARTs. A ctDNA methylation panel of 6 markers revealed 17 times more chances of relapse in ctDNA-positive patients when evaluated after 1 month of surgery in stage I–III CRC (Mo et al., 2023). The same study followed patients who received adjuvant chemotherapy and found that ctDNA positivity led to shorter recurrence-free survival (Mo et al., 2023), highlighting that continuous ctDNA monitoring can help in accessing disease progression and therapy response. In patients prescribed for ACT, post-operative ctDNA positivity is associated with a risk of relapse, compared to patients responding as negative for ctDNA during ACT cycles (Henriksen et al., 2022). The serial assessment of ctDNA followed through post-operative and ACT cycles indicated better chances of survival and a reduced risk of relapse in the reduced ctDNA growth rate over time (Henriksen et al., 2022). On the completion of definitive therapy (surgery or adjuvant therapy) in stage I–IV CRC patients, the assessment of ctDNA for minimal residual disease (MRD) helped in predicting a 100% recurrence in the ctDNA-positive group versus 24% in those without any detectable ctDNA. This method of ctDNA assessment over 1-year follow-up after definitive therapy represented 55% sensitivity, which was enriched to 69% when genomic alteration and epigenomic markers were considered (Parikh et al., 2021). The above study also showcases the importance of epigenomic markers of ctDNA having more sensitivity for recurrence prediction than plasma CEA, reiterating the importance of consistent longitudinal surveillance using an inclusive biomarker panel of ctDNA, epigenomic, and genomic mutational markers (Figure 4).

In another milestone innovation, a blood-based assay, ColonAiQ, was developed after narrowing down 108 methylation markers to only 6 strong predictive markers in ctDNA using a specialized algorithm and designing these 6 biomarkers as an assay. This assay could predict relapse in 85% of cases that had a positive ctDNA methylation status after definitive therapies (surgery or adjuvant therapies), which persisted throughout follow-up (Cai et al., 2021). In the case of gastric cancer, a 38-gene panel to detect somatic mutations in ctDNA was found to predict an increased risk of recurrence and poor survival in patients found positive for these mutations (Varkalaite et al., 2021). More recently, various ctDNA-related features, such as blood tumor mutational burden (bTMB) and CNVs, have been investigated for clinical utilities. In a similar study with GC patients receiving nivolumab monotherapy, a higher blood tumor mutational burden (>6 mt/mb) was associated with better overall survival and longer PFS, whereas a reduced bTMB was found to confer a better disease control rate (DCR). However, the study also noted that the best survival among the cohort was registered by those with higher TMB and a negative CNV in ctDNA assessments (Inagaki et al., 2023).

In an ongoing study, the identification of novel genomic alterations in cfDNA could hold actionable therapeutic interest, such as ERBB2, FGFR2, and TP53 in 4.9%, 6.2%, and 38.3%, respectively. The identification of these mutations could distinguish GC cases from others (Kim et al., 2024). In PDAC patients, the Microsatellite Instability (MSI)-high status is usually assessed for therapy using immune checkpoint inhibitor drugs like pembrolizumab, nivolumab, or those administered with ipilimumab. The use of liquid biopsy to assess this MSI-H detection in cfDNA expectedly predicted better outcomes in immune checkpoint inhibitors as with conventional testing using tumor tissues (Chakrabarti et al., 2022). In resectable PDAC cases, the assessment of KRAS mutations greatly affects treatment outcomes. A multiplex KRAS detection tool based on cfDNA could find a strong association between the mutational concentration of KRAS and its fractional abundance to effectively determine progression-free survival (Kim et al., 2018). Detectable ctDNA is associated with shorter progression-free survival, whereas KRAS mutant allele fractions detected in ctDNA and exosomal DNA were found to be strong predictors of overall survival and PFS (Bernard et al., 2019). In unresectable PDACs that are registered for first-line chemotherapy, the positive assessment of KRAS mutations in ctDNA had unfavorable responses to chemotherapy and, thus, could help in the early detection of patients not expected to gain favorable treatment outcomes (Watanabe et al., 2023). However, it is imperative to continue developing more sensitive methods that can improve the identification of ctDNA originating from tumors and also increase its prognostic prediction accuracy through the inclusion of epigenetic, genomic, and other blood-based biomarkers (Cao et al., 2020).

## 7 Exosomes and exosome-derived biomolecules

Exosomes are released by all cells of the body. They are extracellular vesicles of approximately 149 nm in size, which represents the phenotypic characteristics of the cells from where they were generated; hence, they are prominently heterogeneous (Kalluri and LeBleu, 2020). Like other sub-cellular components, exosomes are also built from the lipid bilayer and also possess nucleic acids such as DNA, RNA, miRNAs, lncRNAs, tRNAs, snRNAs, and circRNAs, which can act as useful biomarkers. An average healthy human blood sample contains ∼1,900 trillion exosomes, whereas patients with cancer have ∼3,800 trillion exosomes (Doyle and Wang, 2019). The reason for the increase in exosome diversity is not well known; however, it is assumed that due to reformed cellular physiology, exosome populations increase in density and diversity (Zhang X. et al., 2019; Gurung et al., 2021; Dilsiz, 2022). Exosomes are considered more promising biomarkers and targets for therapy as they are found in greater numbers and remain stable in circulation. Exosomes have a dual nature in tumor progression and were shown to possess antitumor functions to prevent disease progression (Zhang Y. et al., 2019; Doyle and Wang, 2019). Exosomes can be detected in many biological secretions, like blood, urine, saliva, tears, breast milk, amniotic fluid, and cerebrospinal fluid. The frequent identification of exosomes in secretion makes it efficient for the diagnosis of malignancies. It is also majorly found in pancreatic, ovarian, and breast cancer patients (Zhang H. et al., 2019; Osaki and Okada, 2019; Paskeh et al., 2022), among others.

### 7.1 Exosome miRNAs for the clinical management of GI cancers

miRNAs are mainly short-length RNA species with strand lengths of approximately 19–21 nucleotides, which are found to regulate tumor pathogenesis and progression as tumor suppressors or initiators through several mechanisms (O’Brien et al., 2018). miRNAs play a major role in cancer progression by interacting with tumor-to-stromal interactions, immune invasion, angiogenesis, and the tumor microenvironment (Peng and Croce, 2016; O’Brien et al., 2018). These miRNAs have multiple clinical utilities, from being used as a tool for molecular diagnosis to a method for the prognosis assessment of several cancers (Galvão-Lima et al., 2021; Mondal et al., 2023). These miRNAs can become associated with exosomes as miRNAs shredded from tissue damage or programmed cell death can enter the bloodstream via micro-vesicles and exosomes by binding to HDL, AGO2, and LDL proteins (Li Z. et al., 2022). Exosomal miRNAs play a crucial role in all three aspects of identifying potential markers for diagnosis, prognosis, and predicting OS and DFS and the therapy for chemosensitive or resistant tumors (Cao et al., 2022). Certain miRNAs have high expression in the serum exosomes of CRC patients, such as miR-21, miR-1229, miR-150, let-7a, miR-223, miR-23a, and miR-1246. These findings suggest the origin of tumors in colonic tissue and can help in the differential diagnosis of CRC (Ogata-Kawata et al., 2014). Exosome miRNAs were also reported to have improved the diagnosis of early-stage CRC. For instance, miR-125a-3p, when combined with the CEA marker, suggests early-stage CRC with an AUC curve of 0.855 (Wang et al., 2017). CRC stages I–II were reported to have elevated levels of exo-miR-1246, especially a noted expression in stage II, underlining its early-stage association compared to healthy individuals (Ogata-Kawata et al., 2014; Wang et al., 2017). An elevated level of miR-21 is reported to persist in colonic adenoma throughout advanced CRC stages, indicating its diagnostic importance (Uratani et al., 2016). However, the lone use of a single miRNA would be less specific for diagnosing any cancer type as miRNAs are also elevated in other malignancies. Therefore, the use of a panel of exosome-associated miRNAs has yielded better diagnostic and predictive potential (Uratani et al., 2016).

A tested miRNA panel consisting of miR-21 and miR-1246 was combined with a few other miRNAs (miR-23a, miR-1229, miR-150, miR-223, and let-7a) for increasing diagnostic efficacy for various CRC stages (Uratani et al., 2016). Exosomal levels of this miRNA panel can help in cancer surveillance as their concentration is reported to reduce after resectional surgery (Ogata-Kawata et al., 2014). Another study demonstrated that the enrichment of miRNA panels in exosomes is better than that of circulating miRNAs. Particular exosomal miRNA panels consisting of exo-miR-23, exo-miR-16, and exo-let-7 were strongly enhanced in the CRC patient group compared to healthy individuals (Dohmen et al., 2022). Some miRNAs can have clear expressional differences in various stages of disease, which can be exploited for stage-specific diagnosis. Another miRNA, miR-320b, was found to be enhanced in stage IV CRC and not in stages I–II CRC. Similarly, miR-320d is also found to be highly expressed in metastatic CRC (Tang et al., 2019). In gastric cancer as well, exo-miR-590-5p had elevated expression in the lower stages (stage I/II) and reduced expression in the advanced stages (stage IV) and metastatic CRC (Zheng G.-D. et al., 2021).

Some exosome miRNAs can also predict survival chances, recurrence, and therapy response. Among these, a reduced level of miR-422-3p is related to higher chances of recurrence in stage I/II CRC individuals (Liu C. et al., 2016). However, its role in determining the FOLFOX adjuvant therapy response in progressive CRC stages remains elusive. In gastric cancer, elevated levels of exosomal miRNA miR-590-5p were found to be responsible for decreased overall survival (Zheng B. et al., 2021). Yet another exosomal miRNA exo-miR-92a derived from GC patients was found to play a multifaceted role where its high levels could signify shorter survival, metastatic state, and poor survival outcomes (Yang et al., 2020). A meta-analysis of multiple such studies on miR-92a underlines its important role in prognosis (Guo et al., 2021). Poor survival and recurrence risk (peritoneal recurrence-free survival) increase with high expression of exosome-encapsulated exo-miR-21 in GC cases, as reported in a study (Soeda et al., 2019). Likewise, a range of exo-miRNAs are found to have prognostic and therapeutic importance. An elevated exo-miR-215-5p level is associated with poor DFS and has prognostic utilities in HCC. For instance, exo-miR-10b-5p can be used as a biomarker to identify the early stages of this disease (Cho et al., 2020). miR-720 was found to be consistently elevated in HCC patients compared to patients with other liver diseases. Moreover, exo-miRNAs are also closely associated with small HCCs and intrahepatic tumor stage progression (Jang et al., 2022). Tumor-suppressing exo-miR-199a-3p is found to reverse and control resistance to chemotherapeutic drugs like cisplatin when externally administered (Lou et al., 2020; Zhang et al., 2020). Similarly, many oncogenic miRNAs, such as miR-155 and miR-21, in plasma have been associated with poor survival and progression (Ratnasari et al., 2022), but the roles of their exosomal variants in HCC remain to be explored (Zhou et al., 2018). In the diagnosis and prognosis of pancreatic cancer, exo-miRNAs might be employed, such as exo-miR-1226, which is usually downregulated in PDAC cases compared to benign lesions of the pancreas. Its elevated levels in PDAC patients may signify higher invasiveness and a higher risk of metastasis and recurrence of this disease (Wang F.-W. et al., 2021). On the other hand, exo-miRNAs enriched in portal blood rather than peripheral blood, such as exo-miR-21, exo-miR-451a, and exo-miR-4525, can be employed for identifying PDAC cases undergoing resectable surgery. These exo-miRNAs can be utilized for assessing cases with a high risk of recurrence and poor survival (Kawamura et al., 2019). When considering an efficient exo-miRNA panel, a study identified an exosome miRNA signature having six exo-miRNAs (miR-1273f, miR-1229-3p, miR-432-5p, miR-195-5p, miR-133a-3p, and miR-130b-5p), a higher risk score of which could predict higher chances of recurrence and poor survival outcomes in PDAC cases (Nishiwada et al., 2022).

### 7.2 Exosome lncRNAs

Long non-coding RNAs (lncRNAs) are non-protein-encoding RNA transcripts, usually longer than 200 nucleotides and are sometimes termed “mRNA-like” because of their plausibility to acquire RNA-like modifications such as polyadenylation, 7-methylguanosine capping, or splicing (Mattick et al., 2023). Tumor-suppressor lncRNAs and oncogenic lncRNAs are the two types of lncRNAs that can be distinguished based on their roles in tumors (Guzel et al., 2020). Moreover, in recent years, studies have identified lncRNAs linked to cancer in blood or other bodily fluids that are comparatively stable (Beylerli et al., 2022). lncRNAs found in plasma or serum may be employed as possible biomarkers for various tumor types (Le et al., 2021; Beylerli et al., 2022). Few studies have examined exosomal lncRNA expression as a possible minimally invasive diagnostic biomarker in various gastrointestinal cancers, such as colorectal cancer, gastric cancer, and pancreatic cancer (Gao et al., 2020; Kumar et al., 2022). A growing body of research has revealed that circulating lncRNAs are implicated in various gastrointestinal diseases and conditions (Liu et al., 2022). These include lncRNAs upregulated in hepatocellular carcinoma (highly upregulated in liver cancer, HULC) or in colorectal cancer (colon cancer-associated transcripts, CCATs) and various others (Zhang X. et al., 2019; Liu et al., 2022; Liau et al., 2023). Some of the lncRNAs that are upregulated in CRC are LNCV6/116109, LNCV6/98602, LNCV6/98390, LNCV6/84003, LNCV6/38772, and LNCV/108266 (Chen and Shen, 2020; Preethi et al., 2022).

In CRC management, the expression of exosome miRNAs such as HOTTIP (Oehme et al., 2019), SPINT1-AS1 (Li et al., 2018), and RPPH1 (Liang et al., 2019) can be determined to assess various survival parameters. Few other exosome miRNAs, such as GAS5 (Xu J. et al., 2020), H19 (Ren et al., 2018), 91H (Gao et al., 2018), and RPPH1 (Liang et al., 2019), correlate with the TNM stage, whereas others, such as HOTTIP (Oehme et al., 2019), CRNDE-h/p (Liu T. et al., 2016; Yu et al., 2017), APC1 (Wang J. et al., 2021), and CCAT2 (Wang S. et al., 2019), are reported to influence distance or lymph node metastasis. In gastric cancers, similar dysregulated exosomal lncRNAs could provide clinically valuable clues. Like an exosomal lncRNA, FRLnc1 is reported to signify poor survival and chances of recurrence and can also represent lymph node and distant metastasis (Kurnick et al., 2019; Zhang et al., 2021). There are also a wide range of exosomal miRNAs that potentially have diagnostic and prognostic applications in various gastrointestinal cancers (Gao et al., 2018; Roshani et al., 2022).

Moreover, exosomal lncRNAs have been shown in recent research to facilitate cell-to-cell communication within the TME, which aids in the growth and chemoresistance of cancer cells. For instance, by triggering the Wnt pathway, the overexpression of CAF-derived exosomal H19 can improve chemoresistance and encourage the stemness of CRC cells (Ren et al., 2018). Furthermore, exosomal colorectal cancer-associated lncRNA (CCAL) has been demonstrated to directly connect with the mRNA-stabilizing protein human antigen R (HuR), thereby enhancing CRC cell resistance to oxaliplatin (Deng et al., 2020). Consequently, as they might be crucial to the development of tumors, it is worth investigating the new lncRNAs that are abundant in exosomes created by endothelial cells that surround the TME. Exosomal lncRNA-UCA1 has been shown by Yang et al. to be capable of transmitting cetuximab resistance to susceptible CRC cells, and its expression is strongly associated with cetuximab treatment in CRC patients (Luan et al., 2020). Chen et al. (2020a) found that exosomal HOTTIP is significantly expressed in CRC cells that are resistant to mitomycin and can enhance CRC resistance to mitomycin by preventing miR-214 from degrading KPNA3. Therefore, one intriguing strategy for treating chemoresistance in CRC may be to target exosomal lncRNAs.

### 7.3 Exosomal circRNAs

The size of cirRNAs in exosomes ranges from 201 to 599 bp. circRNAs are a part of endogenous lncRNAs that have closed loops and are stable with longer half-lives (Wang Y. et al., 2019). One of the important functions of cirRNAs is to bind with RNA or proteins to regulate alternative splicing and transcription (Wang H. et al., 2019; Xu Y. et al., 2020). Dysregulated circRNAs and their contribution to influencing various malignancies have been well researched, with potential for clinical applications (Li et al., 2015; Zhang et al., 2023). Exosome-encapsulated circRNAs are found to be more stable in the serum/plasma of patients and, hence, more likely to indicate accurate lncRNA-associated clinical insights than other free circRNA species (Li et al., 2015). These molecular species can also be exploited for critical clinical information pertaining to prognosis and therapy responses (Yi et al., 2023). In CRC patients, exosomal has_circ_0004771 is upregulated compared to the serum of CRC post-operative patients, thereby showing potential as an early-stage detection biomarker for CRC (Pan et al., 2019). One such circulating exosome, circ-FBXW7, in combination with miR-128-3p, contributes to chemoresistance for oxaliplatin in CRC, thus suggesting an important therapeutic marker for CRC patients (Xu et al., 2021; Xu et al., 2024). Additionally, many exosomal circRNAs were found to play an important role in determining the resistance to chemotherapy in many cancer types, like gastric, esophageal, colorectal, pancreatic, and non-small lung cancers (Xu et al., 2024). However, a thorough mechanism remains unknown due to limited studies.

### 7.4 Exosomal proteins

Exosomal proteins are gaining interest in the detection of plasma and serum circulation. The transfer of oncoproteins from intercellular exosomes helps in the progression of tumors (Li W. et al., 2017), and thus, these exosomal proteins may carry tumor-specific biomarkers into circulation and can be used for diagnostic and prognostic applications (Wang X. et al., 2022; Liu et al., 2023). These proteins reflect the cellular origin of exosomes and also contribute to the detection of cancer. In pancreatic cancer, exosome proteins are reported to be in an elevated form as macrophage migration inhibitory factor (MIF) compared with healthy subjects (Chang and Pauklin, 2021). Moreover, the cell surface proteoglycan glypican-1 (GPC-1) is also found in exosomes extracted from pancreatic and breast cancer patients (Lorenzon and Blandino, 2016; Wang et al., 2019b), which can also be detected in the early and late stages of pancreatic cancer compared to the serum of healthy controls. In CRC patients, low CPNE3 exosomal proteins have shown better OS and DFS than higher CPNE3 exosomal protein levels in healthy individuals (Sun et al., 2019; Sun et al., 2019). In contrast, the QSOX1 exosomal protein is found to be low in the plasma of CRC patients compared to controls, potentially revealing diagnostic biomarkers with an ROC curve (AUC of 0.904) (Ganig et al., 2021). Furthermore, a possible therapeutic strategy for CRC would be to target the exosomal prion proteins found to be overexpressed in CRC. Drugs targeting these prion proteins, when administered along with 5-Fu, yielded better suppression of CRC progression (Yun et al., 2021). Moreover, exosomal PD-L1 is expressed in higher concentrations by cancer cells than that by healthy cells, and hence, its presence could indicate poorer prognosis (Lawler et al., 2020; Hu et al., 2023) (Table 3).

## 8 Technical challenges regarding the translation of liquid biopsy

Many clinical trials testing the efficiency of liquid biopsy components in the diagnosis of GI tract cancers have been conducted globally (Table 4). Despite that, the translation of liquid biopsy for clinical applications in cancer will require multiple challenges to be addressed. Among these, a major role is played by improving cellular and sub-cellular analyte isolation efficiency using better enrichment protocols (Li et al., 2020). Usually, many sequencing technologies are used to detect ctDNA, aiming to detect rare mutations and compare them with wildtype sequences. The most important recent techniques for the analysis of liquid biopsy are PCR-based and NGS technology. The digital droplet PCR (ddPCR) can identify tumor-specific mutations with high accuracy. ddPCR is a sensitive and rapid technology suitable for detecting mutations, and many such assays are commercialized, but it is limited by the restricted number of probes for each reaction. However, these limitations can be overcome with NGS platforms, which provide whole-genome sequencing of even new and rare molecular alterations without any prior knowledge (Lin S. Y. et al., 2021). NGS is also used to determine somatic single-nucleotide variants, allelic frequencies, copy number variations, and DNA methylation for ctDNA in plasma (Lin C. et al., 2021). Targeted sequencing of desired regions can also be performed using NGS platforms, but it requires a high ctDNA concentration for definitive data, indicating genome-wide changes specific to the tumor. Therefore, targeted sequencing has low specificity (Glenn, 2011), which can be improved by adapting to several methods to reduce background error rates (Snyder et al., 2016). Furthermore, bioinformatics-based approaches can be used to improvise the method and reduce error rates (Ma et al., 2019). One such tool used is the base position error rate (BPER), which is an algorithm used to detect the mean of all types of errors caused by the sequencing environment (Pécuchet et al., 2016). Another drawback of isolating and analyzing cfDNA/ctDNA from cancer patients is its disturbed integrity due to the presence of numerous ultrashort and jagged DNA fragments within ctDNA. This problem has been addressed by using a modified NGS pipeline tool known as BRcfDNA-Seq, which allows the faithful analysis of such low-integrity ctDNA in body fluids (Swarup et al., 2023).

**TABLE 4:** Some clinical trials pertaining to liquid biopsy analyses in GI tract cancer.

Trial	Subjects	Findings	Reference
CAPRI-GOIM trial	340 mCRC patients with KRAS exon-2 wildtype phenotype	RAS testing in tissues and peripheral blood liquid biopsy predicts similar outcomes	Normanno et al. (2018)
PROSPECT-C phase II CRC trial	47 RAS wildtype CRC patients	Longitudinal liquid biopsy assessment of RAS mutations is useful for predicting primary and acquired resistance to anti-EGFR therapies	Khan et al. (2018)
Translational biomarker phase II pilot study	30 mCRC subjects	Higher ctDNA levels associated with the metastatic status of CRC and can predict survival among patients receiving regorafenib treatment	Unseld et al. (2021)
CAIRO5 clinical trial	183 mCRC subjects	Longitudinal assessment of ctDNA was more predictive of overall survival than radiological response analysis	van ’t Erve et al. (2023)
JACCRO CC-11	68 CRC subjects receiving FOLFOXIRI treatment	RAS mutation clearance during treatment course associated with longer overall survival	Sunakawa et al. (2022)
SORAMIC trial	13 subjects with unresectable advanced HCC	cfDNA levels and ctDNA mutations (BAX, HNF1A, and CYP2B6 gene mutations) correlated with metastases and survival	Alunni-Fabbroni et al. (2019)
Freiburg University Medical Center study	25 resectable PDAC subjects	KRAS mutation and C1-19-9 in plasma associated with poor overall survival and early relapse	Hussung et al. (2021)

CRC, colorectal cancer; GC, gastric cancer; HCC, hepatocellular carcinoma; PDAC, pancreatic ductal adenocarcinoma.

Another such liquid biopsy analyte that emerged as a strong candidate to reveal disease status in cancer patients is exosomes and exosome-derived biomolecules. There are many methods for exosome isolation, each with its own limitations and advantages. The most common methods are ultracentrifugation and precipitation. High-speed centrifugation separates the molecules based on size and density differences. This technique has the potential for the isolation of exosomes. However, the instrument cost, low yield, stability, and large sample requirement make it inappropriate for use in clinical applications. Density gradient centrifugation can also be used with UC to purify exosomes. However, methods such as precipitation can be utilized, which reduces the solubility of exosomes in addition to its lower sample volume requirements and is comparatively inexpensive for clinical utilities. Moreover, new methods of enhanced exosome isolation assisted by microfluidics (Contreras-Naranjo et al., 2017) or nanomaterials (Fang et al., 2022) can be explored for higher isolation efficacies. In addition to the above, another technical challenge would be to reduce the false-positive scoring of analytes due to the presence of other conditions, such as stress, inflammatory disease, infectious disease, and other co-morbidities that are unrelated to tumors, so as to score only tumor-associated molecular or cellular footprints using liquid biopsy.

## 9 Conclusion

Liquid biopsy utilizes a range of tumor-related cellular, sub-cellular, and molecular analytes. These analytes are enumerated, qualified, quantified, and scored using varied parameters for use in several cancer types. These parameters are recorded using many technical and analytical tools on many platforms, ranging from flow cytometry, PCR, ddPCR, and immunofluorescent imaging to next-generation sequencing platforms. These techniques capture these analytes at different levels of sensitivities, given that the proportions of these disease-associated analytes are rare and comparatively limited in the bodily fluids from which they are isolated. Therefore, one of the major challenges to scoring these analytes before translating them into clinical settings would be to standardize them in large cohort studies. Moreover, another challenge of limited sensitivities and specificities of an analyte can be addressed by using a multi-analyte study design comprising a panel of biomarkers or a combination of various liquid biopsy analytes. For instance, a study observed an increase in the sensitivity of a singular miRNA-based test when it was co-analyzed with a serum biochemical marker CEA in detecting early-stage CRC (Wang et al., 2017). Similar analyses using top-scoring analytes could yield better and more focused sensitivities to diagnose a disease condition. Another consideration could be to ensure enhanced repeatability and dependability of outcomes through standardized protocols and processes that are required across various research and clinical settings across the world so that results can be compared without any false conclusions. Moreover, the detection of rare mutations and methylation markers or CNVs in molecular species such as ctDNA or cfDNA are better indicators of tumor progression and therapy response and, hence, must be further explored (Wu et al., 2021; Mo et al., 2023). Apart from this, liquid biopsy can have immense potential to emerge as a screening tool to identify early occurrences of cancer in asymptomatic and at-risk patients. This would require large-scale multi-center studies with consideration for multi-analyte investigations to decrease statistical biases with patient and biomarker diversity.
